# The Effect of Intensive Close-Kinetic-Chain Exercises on Functionality and Balance Confidence After Total Knee Arthroplasty

**DOI:** 10.7759/cureus.18965

**Published:** 2021-10-22

**Authors:** Theano Thonga, Sophia Stasi, George Papathanasiou

**Affiliations:** 1 Physiotherapy and Paramedical Department, General Hospital of Attica “KAT”, Athens, GRC; 2 Laboratory of Neuromuscular and Cardiovascular Study of Motion - LANECASM, Physiotherapy Department, University of West Attica, Athens, GRC

**Keywords:** balance confidence, functionality, close kinetic chain exercise, postoperative physiotherapy intervention, primary total knee arthroplasty

## Abstract

Objectives

The aim of this study was to evaluate the effects of an additional close-kinetic-chain exercise program (CKC-PT), in conjunction with the standard physiotherapy intervention (TKA-PT), on the general health status, functionality, balance conﬁdence, and postoperative falls of knee osteoarthritic patients who had undergone total knee arthroplasty (TKA).

Patients and methods

Thirty community-dwellers, aged >65 years, were randomized into equal groups. The Greek versions of the SF-36 version1.0 (SF-36v1.0-Gr), WOMAC^®^ (WOMAC^®^-Gr), Activities-speciﬁc Balance Conﬁdence scale (ABC-Greek), Timed Up and Go (TUG) test, and Berg Balance Scale were assessed preoperatively and twice postoperatively (7^th^ week and 12^th^ month). Non-parametric (Mann-Whitney test) and parametric (two-way analysis of variance (ANOVA) model and student t-test) analyses were used to compare the percentage changes in all variables.

Results

The CKC-PT group reported better (%) functional improvement (WOMAC^®^-Gr Physical­ Function subscale) and higher (%) balance confidence (ABS-Greek) at the seventh week and twelfth month as compared to TKA-PT (p<0.05). No other statistically significant differences were observed.

Conclusions

The implementation of a close-kinetic-chain exercise program, in addition to standard physiotherapy, may significantly increase both the functionality and balance conﬁdence of patients who have undergone TKA. Further studies are needed to verify these findings.

## Introduction

Over the past four decades, total knee arthroplasty (TKA) has become the most successful surgery for patients with severe knee osteoarthritis (OA) [1]. Patients who undergo TKA show marked improvements in function, and a reduction in pain compared with their preoperative limitations and symptoms [2]. In addition, postoperative physiotherapy greatly influences the short- and long-term functional outcomes after TKA [1-2]. Although post-TKA standard physiotherapy programs may vary across various rehabilitation settings, they typically focus on pain and edema management, regaining the knee joint’s range of motion, lower extremity strength and a normal gait pattern, and on functional activity training [2].

However, it has been reported that the decreased knee extension strength, functional ability, and proprioception, as well as gait disorders that are observed preoperatively in knee OA patients sometimes deteriorate further postoperatively and may increase the risk of falls [3-4]. The lower limb proprioception deficiency has been reported to persist for one year following TKA, despite improvements in knee extension strength [5]. As the authors pointed out, this may be explained by the fact that during the surgical procedure, in order to restore the joint spaces damaged by the OA and the intraarticular geometry, some of the knee’s ligaments are removed or released, resulting in a lack of proprioceptive ability [5]. Studies have identiﬁed deﬁcits in components of the balance system, such as decreased ability to detect joint position and motion, delayed muscle latency, altered amplitude of muscle activity, and decreased postural control, in patients after TKA [5-6]. Therefore, exercises aimed at improving the impaired movement control and balance of patients after TKA should be considered [5].

Close-kinetic-chain (CKC) is an effective type of exercise for improving balance ability [7]. Performed with partial or full compressive loads, it effectively stimulates the proprioceptive system by proprioceptive feedback to initiate and control muscle activation patterns [7]. In addition, CKC exercises are performed in functional positions and include concentric, eccentric, or isometric muscular activity. In addition to muscular co-activity, these exercises also load non-contractile soft tissues such as ligaments, tendons, and joint capsules. Therefore, they improve muscular strength and power, stability, balance, and synergy in functional loading positions [8].

The main purpose of the present trial was to study the effects of an additional CKC exercise program, in conjunction with the standard physiotherapy intervention, over a 12-month follow-up period, on the general health status and functional ability of patients with severe knee OA who had undergone TKA. Additionally, it was investigated whether this combined physiotherapy intervention would influence balance conﬁdence or the incidence of postoperative falls.

## Materials and methods

Design and ethical aspects of the study

This interventional clinical trial is part of a larger prospective study investigating the preoperative history and frequency of falls, including the mechanism and/or causes of falls, and the postoperative reduction of falls, in patients with severe knee OA before and one year after TKA [1,9-11]. The present trial was conducted in accordance with the ethical principles stated in the Declaration of Helsinki and its later amendments [12]. The Scientific Research Council of the “Sismanoglio-Amalia Fleming” General Hospital of Attica, Athens, Greece approved the protocol of the study (Ref.: 1882/25-02-2010).

Sample size estimation

In the present study, participants were divided into equally numbered groups because it has been reported that unequal sample sizes can affect the homogeneity of variance assumption between compared groups [13].

It was calculated that a sample size of 30 evaluable patients (15 per group) was required in order to have an 80% probability of demonstrating a between-groups difference of 10% (control group: 10%±9 vs. experimental group: 20%±9) in the percentage change from baseline to 12 months of the Activities-specific Balance Confidence Scale (ABC) score with a significance of<5% (two-tailed test). Estimation of sample size was performed using the G*Power 3.1.9.2 program [14].

Randomization

To be eligible for randomization, patients had to meet the following inclusion criteria: urban community dwellers, aged 65 years or older, with severe knee OA (grade 3 or 4 according to the Kellgren-Lawrence classification system), suffering from knee pain for at least one year and scheduled to undergo TKA [15]. Participants who had lower-limb muscle weakness of central or peripheral neurological etiology or a history of fainting were excluded, as were those taking medication that adversely affected their postural or dynamic balance. Every patient who fulfilled the inclusion criteria was invited to participate in the study. Upon acceptance and prior to surgery, participants gave their written informed consent, and their demographic and clinical characteristics and fall history (falls within the previous 12 months) were recorded during a personal interview. The definition of the Kellogg Group was used to analyze the incidence of falls: “A fall is an event which results in a person coming to rest inadvertently on the ground or other lower level and other than as a consequence of the following: sustaining a violent blow, loss of consciousness, sudden onset of paralysis, as in a stroke or an epileptic seizure’’ [16].

A simple random method with a 1:1 allocation ratio was used for randomization: namely, the first selected patient was assigned to the control group, the second to the experimental group, the third to the control group, and so forth [17]. The randomization procedure was performed by an independent clinician (PS) and the participants were not blinded to group allocation.

Interventions

All participants underwent hybrid total knee arthroplasty through a medial parapatellar approach [18]. All operative procedures were performed by the same team of orthopedic surgeons and the implanted prostheses had similar specifications and technical characteristics. For both study groups, the physiotherapy intervention was initiated on the first postoperative day and continued for seven weeks, on an in-patient basis for one week and home-based for the remaining six weeks. A physiotherapist (TT) member of the research team provided individual sessions to all participants during hospitalization. Following discharge, the rehabilitation program continued at home, under the supervision of the same physiotherapist, until the end of the seventh postoperative week.

**Figure 1:**
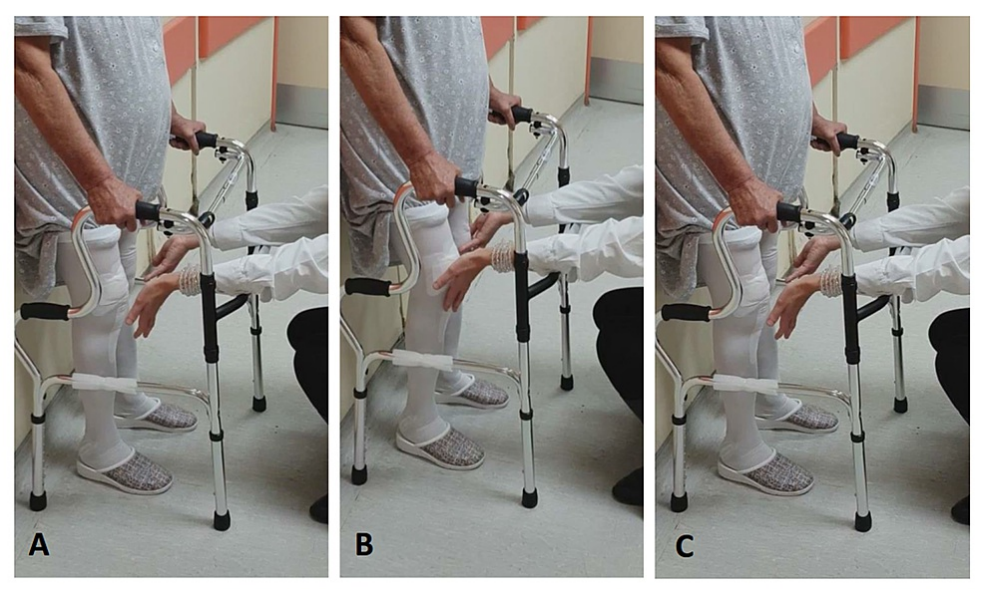
Semi-squat I exercise A: Upright position. The patient stands with the lower limbs positioned in slight hip abduction and the back supported against the wall. The walking device is placed in front to provide a handhold for support. B: The patient bends his/her hips and knees at 30°. C: The patient extends both his/her lower limbs, returning to the initial position. Initially, the load on the operated limb should be a maximum of 30% vs. 70% on the contralateral limb. By the end of the second week, the patient should be able to perform the exercise by gradually maximizing the load on the operated limb up to 70% vs. 30% on the contralateral.

The standard physiotherapy program (TKA-PT) was implemented in the control group, based on the rehabilitation protocol of Papathanasiou et al. (Appendix) [19]. Initially, and up to the fourth postoperative day, the physiotherapy intervention was the same for both study groups. However, from the fifth postoperative day, an intensive, additional close-kinetic-chain exercise program (CKC-PT) was implemented in the experimental group, in conjunction with the standard program, as follows:

1. The additional CKC-PT program was applied twice per day, in the morning after the TKA-PT program and in the evening before the patient’s prescribed ambulation. The rationale for this decision was that the twice-per-day performance could help patients exercise intensely with the CKC-PT program without overloading the operated joint. During the first two sessions (5th and 6th postoperative days), the physiotherapist educated the patient on the proper performance of the exercises. The aim was for patients to emphasize and to fully focus on the sense of motion of the knee joint and control it throughout the implementation of the exercise and to learn to remain in the final position for five seconds by balancing their body weight on the middle of the sole (Figure 1).

2. The duration of the standard physiotherapy program (TKA-PT) was initially 25 minutes, increasing to a maximum of 40 minutes. The additional CKC-PT program added a maximum of 15 minutes per session in the morning while the evening CKC session was performed after a five-minute warm-up.

3. The progression mode of CKC exercises and instructions for their performance are described in Table 1.

**Table 1:** The additional close-kinetic-chain exercise program during the intervention period

Exercise	Performance’s Instructions
5^th^ day - 15^th^ day (2 sessions per day - 5 days per week)	
Bodyweight transfers I	Upright position. The patient stands with the lower limbs positioned in slight hip abduction and the back supported against the wall. The walking device is placed in front to provide a handhold for support. Initially, the load on the operated limb should be a maximum of 30% vs. 70% on the contralateral limb. By the end of the 2^nd^ week, the patient should be able to perform the exercise by gradually maximizing the load on the operated limb up to 70% vs. 30% on the contralateral. Once the patient becomes familiar with the proper performance of the exercise, then in the ending position the patient attempts to rock his/her body weight slightly forward and back, while keeping the entire sole in contact with the ground.
Semi-squat I	Upright position (as described above). The patient’s hips and knees are bent at 30°. Initially, the load on the operated limb should be a maximum of 30% vs. 70% on the contralateral limb. By the end of the 2^nd^ week, the patient should be able to perform the exercise by gradually maximizing the load on the operated limb up to 70% vs. 30% on the contralateral. Once the patient becomes familiar with the exercise, then in the ending position the patient attempts to rock his/her body weight slightly forward and back, while keeping the entire sole in contact with the ground.
16^th^ day – 30^th^ day (2 sessions per day - 5 days per week)	
Bodyweight transfers II	Upright position. The exercise is performed without support from the wall, while the patient maintains the correct trunk and pelvic posture. The exercise is performed by loading each lower limb in succession with 90% load vs. 10% (keeping the entire sole of the less heavily loaded foot in contact with the ground). In the ending position, the patient attempts to rock his/her body weight slightly forward and back, while keeping the entire sole of both feet in contact with the ground. As the program progresses the physiotherapist applies resistance to the pelvis during the movement and the patient tries to maintain the ending position for 5 seconds. The exercise is performed a. with open eyes and b. with closed eyes.
Semi-squat II	Upright position. Without being supported by the wall and while maintaining the correct trunk and pelvic posture, the patient bends hips and knees at 30° with an equal load on both limbs (50% vs. 50%). In the ending position, the patient attempts to rock his/her body weight slightly forward and back while keeping the entire sole in contact with the ground.
31^st^ day – 45^th^ day ( 2 sessions per day - 3 days per week)	
Bodyweight transfers III	Upright position. The body weight transfers are performed on a foam surface, following the instructions for bodyweight transfers II.
Semi-squat III	Upright position. The exercise is performed without support from the wall and with closed eyes, while the patient maintains the correct trunk and pelvic posture. The patient bends the hips and knees at 30°, with an equal load on both limbs (50% vs. 50%). The same exercises are performed on a foam surface: a. with open eyes and b. with closed eyes.
Single-leg stance	Upright position. Single-leg stance for each lower limb. The patient tries to maintain the ending position for 5 seconds (a. on a hard surface and b. on a foam surface).
Step up	Upright position. Standing at the foot of a staircase, the patient lifts the operated lower limb to the first step, then moves it back down; then performs the same sequence with the contralateral limb (a. at his/her own pace and b. at a fast pace).

Upon completion of the physiotherapy intervention (end of the 7th postoperative week), patients of both groups were encouraged to continue their late-stage program three times per week until the end of the third postoperative month. All patients received verbal and written instructions and were asked to record the exercises in a page calendar. The participants’ optimal compliance was ensured by personal meetings with the physiotherapist once per fortnight until the end of the third month.

Evaluation

Outcome measures were obtained at three different time points: prior to surgery (baseline), at the end of the seventh postoperative week (post-intervention), and at the end of the twelfth month after surgery (follow-up). Patients’ general health status, functional capacity, and balance confidence were assessed using the reliable and valid Greek versions of the following self-reported questionnaires: Medical Outcomes Study 36-item Short-Form Health Survey version 1.0 (SF36v1.0-Gr); Likert 3.1 format of the Western Ontario & Mc Master Universities Osteoarthritis Index (WOMAC®-Gr); the Activities-speciﬁc Balance Conﬁdence scale (ABC-Greek); and the objective physical performance measures Timed Up and Go (TUG) test, and Berg Balance Scale (BBS-GR) [20-24]. The incidence of postoperative falls (12 months after TKA) was also recorded by monthly telephone communication.

All assessments were carried out by the same examiner (SS), who was not involved in any way with the rehabilitation program and was blinded with respect to the group assignment.

Instruments

Medical Outcomes Study 36-Item Short-Form Health Survey

The Medical Outcomes Study 36-Item Short-Form Health Survey version 1.0 (SF-36v1.0) is widely acknowledged as the gold standard instrument for assessing health status in general and in specific population groups. It is a 36-item self-reported questionnaire that measures eight dimensions of health status (eight subscales). The dimensions of health status examined by the subscales include physical functioning, social functioning, role limitations due to physical problems, role limitations due to emotional problems, mental health, energy/vitality, bodily pain, and general health perception. The subscale item scores are coded, summed, and transformed into a scale from 0 to 100. A score of 0 represents the worst health status, whereas a score of 100 represents the best health status. Two standardized summary scores can also be calculated from the SF-36; the Physical Component Summary (PCS), and the Mental Health Component Summary (MCS) [20].

Western Ontario & Mc Master Universities Osteoarthritis Index

The Western Ontario & Mc Master Universities Osteoarthritis Index (WOMAC®) was developed for measuring the level of pain, joint stiffness, and functional ability in patients with OA. Its 24-item questionnaire is divided into three subscales: WOMAC-pain (5 items, score range 0-20), WOMAC-stiffness (2 items, score range 0-8), and WOMAC-function (17 items, score range 0-68). According to WOMAC®’s user guide (No X), each subscale-item score is normalized to provide a WOMAC-total score of up to 100, with higher scores indicating worse pain, stiffness, and functional limitations [21].

Activities-speciﬁc Balance Conﬁdence scale

The Activities-speciﬁc Balance Conﬁdence scale (ABC) is a 16-item self-report measure of the perceived balance conﬁdence of an individual during the completion of specific ambulatory activities. Participants estimate, on a scale of 0% to 100%, how conﬁdent they are during the performance of various activities such as picking a slipper up off the ﬂoor or walking on a slippery surface without losing their balance. The item scores are then summed and divided by 16 to provide an overall mean balance conﬁdence score [22].

Timed Up and Go Test

The Timed Up and Go (TUG) test was introduced in 1991 by Podsiadlo and Richardson [23]. The TUG test measures the time (in seconds) taken by a participant to stand up from an armed chair with a seat height of 46 cm, walk for 3 m, turn around a cone and return to sit on the same chair. A shorter performance time represents better functionality [23]. In the present study, the participants were instructed to perform the test with a self-selected “comfortable and safe” gait speed and were allowed to use the walking aid on which they depended at the time of measurements.

Berg Balance Scale

The Berg Balance Scale (BBS) is a 56-point scale that was developed to objectively assess the level of function and balance. The patient is evaluated and graded on a sequence of 14 balance activities (tasks). Scoring for each task ranges from 0 to 4. A score of 0 indicates that the patient is unable to complete a particular task. A score of 4 indicates that the patient can completely carry out the task [24].

Statistical analysis

All analyses were carried out using the Statistical Package for the Social Sciences (SPSS) v 17.00 (SPSS Inc., Chicago, III). All tests were two-sided; a p-value of<0.05 was used to denote statistical significance.

Data were expressed as mean±SD for quantitative variables and as percentages (%) for qualitative variables. The Kolmogorov-Smirnov test was utilized for normality analysis of the quantitative variables.

The comparison of the percentage changes in variables (SF-36v1.0-Gr, WOMAC®-Gr, ABC-Greek, TUG, and BBS-GR) from baseline during the observation period between the two groups was analyzed for non-parametric analysis using the Mann-Whitney test. In addition, the two-way mixed model analysis of variance (ANOVA) for repeated measurements and the student t-test were used for parametric analysis of SF-36v1.0-Gr, WOMAC®-Gr, ABC-Greek, the TUG test, and the BBS test between groups at each time point.

## Results

**Figure 2:**
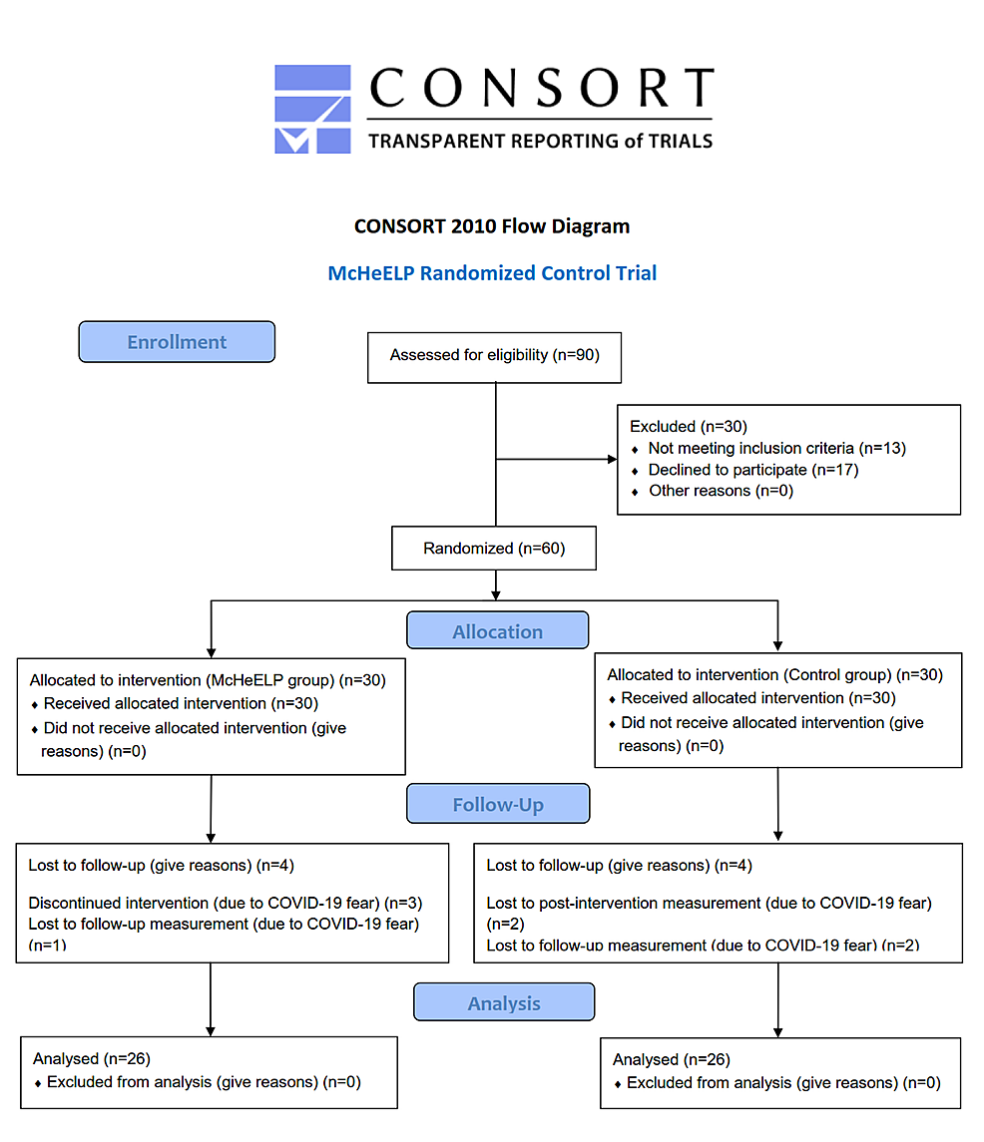
The flow diagram of the study

Patient recruitment lasted until the required number of 15 participants per group had been reached. Initially, 42 patients were invited to participate in the present trial. The recruitment procedure is depicted in the flow diagram in Figure 2.

The patients’ demographic and clinical characteristics at baseline are shown in Table 2. Although most differences between the CKC-PT group and TKA-PT group were non-significant; there was a significant difference in the baseline measurement of the WOMAC®-Gr Physical Function subscale. Therefore, the statistical analysis was carried out by comparing the percentage changes (%) in outcome measures between the two groups at all time points.

**Table 2:** Baseline demographic and clinical characteristics of the participants (n=30) * Values are expressed as mean±SD; SD=standard deviation ^a ^SF-36v1.0-Gr: the Greek version of Medical Outcomes Study 36-Item Short-Form Health Survey; ^b ^WOMAC-Gr: the Greek version of Western Ontario and McMaster Index; ​​​​​​​^c^ ABC-Greek: the Greek version of Activities-speciﬁc Balance Conﬁdence scale; ​​​​​​​^d ^BBS-GR: the Greek version of Berg Balance Scale

Characteristics	CKC-PT Group (n=15)	TKA-PT Group (n=15)	p-value
Age (y)*	71.00±4.37	70.20±3.89	0.601
Body mass index (Kg/m^2^)^*^	29.58±3.88	28.10±4.02	0.313
Sex [n, (%)]			
Men	4 (26.7 %)	5 (33.3%)	0.990
Women	11 (73.3%)	10 (66.7%)	
Pain elsewhere [n, (%)]			
No	13 (86.6%)	10 (66.7%)	0.169
Yes	2 (13.4%)	5 (33.3%)	
Comorbidities [n, (%)]			
1	8 (53.3%)	9 (60.0%)	0.998
2 or 3	7 (46.7%)	6 (40.0%)	
Relatives/social status [n, (%)]			
No	0 (0.0%)	3 (20.0%)	0.224
Yes	15 (100.0%)	12 (80.0%)	
Previous arthroplasty (other joint) [n, (%)]			
No	11 (73.3%)	13 (86.7%)	0.651
Yes	4 (26.7%)	2 (13.3%)	
Falling status [n, (%)] (1 year preoperative)			
Fallers	10 (66.7%)	9 (60%)	0.048
Non-fallers	5 (33.3%)	6(40%)	
Preoperative outcome measures			
SF-36v1.0-Gr _Physical Component Subscale_^*a^	34.65±5.94	37.47±8.07	0.285
SF-36v1.0-Gr _Mental Component Subscale_^*a^	39.43±11.28	34.19±8.23	0.157
WOMAC®-Gr Pain^ * b^	228.33±80.10	215.00±90.04	0.672
WOMAC®-Gr Stiffness^ * b^	66.67±47.22	36.67±41.04	0.110
WOMAC®-Gr Physical Function^* b^	605.00±276.97	368.33±144.07	0.007
ABC-Greek^*c^	67.09±16.22	73.71±15.54	0.263
Timed Up and Go test^*^	11.35±3.11	10.89±2.40	0.652
ΒBS-GR ^* d^	47.07±4.59	47.53±3.83	0.765

The non-parametric Mann-Whitney analysis found statistically significant differences between the CKC-PT and TKA-PT groups only in the percentage changes in WOMAC®-Gr Physical Function and ABC-Greek at the end of the seventh postoperative week (post-intervention measure) (p=0.002 and p=0.045, respectively). This statistically significant difference was preserved in the follow-up measurement at the end of the 12th postoperative month (p=0.021 and p=0.010, respectively) (Table 3).

**Table 3:** Comparison of (%) percentage change of the variables between the two groups (non-parametric analysis using the Mann–Whitney test) * Values are expressed as median ^a ^SF-36v1.0-Gr: the Greek version of Medical Outcomes Study 36-Item Short-Form Health Survey version 1.0; ^b^ WOMAC-Gr: the Greek version of Western Ontario and McMaster Index; ​​​​​​​^c ^ABC-Greek: the Greek version of Activities-speciﬁc Balance Conﬁdence scale; ​​​​​​​^d ^BBS-GR: the Greek version of Berg Balance Scale

Preoperative vs. 6^th^ postoperative week (%)				
Variables		TKA-PT Group	CKC-PT Group	p-value
		SF-36v1.0-Gr_Physical Component Subscale_^ *a^			18.97	19.02	0.902
SF-36v1.0-Gr_Mental Component Subscale_^* a^		13.33	22.91	0.461
WOMAC®-Gr Pain^* b^		-75.00	-83.33	0.775
WOMAC®-Gr Stiffness^ *b^		.00	.00	0.233
WOMAC®-Gr Physical Function^* b^		-23.81	-58.33	0.002
ABC-Greek ^* c^		-8.90	0.00	0.045
Timed Up and Go test^ *^		-9.53	-12.24	0.412
ΒBS-GR ^* ^^d^		6.00	8.16	0.187
Preoperative vs. 1 postoperative year (%)				
Variables	TKA-PT Group		CKC-PT Group	p-value
SF-36v1.0-Gr_Physical Component Subscale_^ *a^	43.97		63.27	0.074
SF-36v1.0-Gr_Mental Component Subscale*_^ a^	25.29		29.65	0.967
WOMAC®-Gr Pain^ *b ^	-100.00		-100.00	0.713
WOMAC®-Gr Stiffness^ *b^	.00		-50.00	0.137
WOMAC®-Gr Physical Function^ *b^	-80.95		-91.67	0.021
ABC-Greek ^*c^	15.09		27.26	0.010
Timed Up and Go test^ *^	-29.39		-32.71	0.406
ΒBS-GR ^*^^d ^	6.00		8.16	0.539

The parametric analysis also found statistically significant differences in the percentage change of the variables WOMAC®-Gr Physical Function and ABC-Greek at the end of the 7th postoperative week (p<0.005 and p=0.041, respectively) (Table 4). In the follow-up measurements, a statistically significant difference was observed in WOMAC®-Gr Physical Function (p=0.038), but not ABC-Greek (p=0.081) (Table 4).

**Table 4:** Comparison of (%) percentage change of the variables between the two groups (parametric analysis using the two-way mixed model ANOVA for repeated measurements and the student t-test) * Values are expressed as median ^a ^SF-36v1.0-Gr: the Greek version of Medical Outcomes Study 36-Item Short-Form Health Survey version 1.0; ^b^ WOMAC®-Gr: the Greek version of Western Ontario and McMaster Index; ​​​​​​​^c^ ABC-Greek: the Greek version of Activities-speciﬁc Balance Conﬁdence scale; ​​​​​​​^d^ BBS-GR: the Greek version of Berg Balance Scale

Preoperative vs 6^th^ postoperative week (%)			
Variables	TKA-PT Group	CKC-PT Group	p-value
SF-36v1.0-Gr_Physical Component Subscale_^*a^	16.19±19.20	18.97±22.01	0.716
SF-36v1.0-Gr_Mental Component Subscale_^*a^	21.60±33.96	31.60±37.43	0.450
WOMAC®-Pain^ *b^	-69.08±32.63	-72.50±24.47	0.747
WOMAC®-Stiffness^ *b^	-5.00±42.47	-26.67±44.79	0.185
WOMAC®-Physical Function^ *b^	-2.17±61.40	-53.36±23.31	<0.005
ABC-Greek ^*c^	-8.34±22.11	9.85±26.54	0.041
Timed Up and Go test^ *^	-8.53±6.53	-10.48±9.44	0.515
ΒBS-GR^ *d^	6.08±3.93	8.65±4.26	0.102
Preoperative vs 1 postoperative year (%)			
Variables	TKA-PT Group	CKC-PT Group	p-value
SF-36v1.0-Gr_Physical Component Subscale_^*a^	54.44±45.46	74.72±34.11	0.178
SF-36v1.0-Gr_Mental Component Subscale_^*a^	31.35±29.41	33.45±36.89	0.864
WOMAC®-Pain^ *b^	-93.69±11.95	-96.72±6.49	0.395
WOMAC®-Stiffness^ *b^	-20.00±46.48	-50.00±53.45	0.112
WOMAC®-Physical Function^ a^	-63.71±42.00	-88.08±10.19	0.038
ABC-Greek ^*c^	20.54±21.86	40.95±37.86	0.081
Timed Up and Go test^ *^	-29.21±8.56	-35.22±9.61	0.081
BBS-GR ^* d^	14.77±4.89	18.48±10.19	0.214

As regards the effect of the combination of the TKA-PT program with the additional CKC-PT compared to the TKA-PT program alone in the reduction of postoperative falls, the results showed that there was no significant statistical difference between the two groups (data not shown).

## Discussion

In the present study, CKC exercise in addition to the TKA-PT intervention was found to increase the postoperative functional ability and balance conﬁdence of patients significantly more than the standard TKA-PT program.

The patients in the CKC-PT group reported both better (%) functional improvement and higher (%) balance confidence (as measured with the WOMAC®-Gr Physical Function subscale and ABS-Greek, respectively) compared with the TKA-PT group. These findings are in line with studies suggesting that CKC exercises provide balance control and increase the self-confidence of older people, improve their functional capacity, and consequently their mobility in the performance of daily-life activities [25]. However, no statistically significant difference between groups was observed in relation to the self-reported SF-36v1.0-Gr or the objective physical performance measures, the TUG test, and ΒBS-GR. The SF-36 findings may be explained by the fact that our sample size estimation was based on the probability of demonstrating a between-groups difference in balance confidence, as expressed by the percentage change of ABC score. This sample size estimation seemed to be adequate for extracting statistically significant differences between groups in patients’ general health status, as expressed by the SF-36v1.0-Gr. The inability to show a statistically significant difference between groups regarding the TUG test and BBG-GR might be explained by the fact that both are objective physical performance measures. The WOMAC® and ABS are self-reported questionnaires that collect information directly from the participants about their skills while performing daily-life activities, so subjects must rate themselves. On the other hand, the TUG test and BBS are physical performance measures that assess functional ability using a different approach. These measures are used by a clinician to evaluate discrete and specific components of the performance of specific tasks, including how the task was approached under “laboratory conditions.” Although the two measurement methods are different, they provide important and complementary information about the patients’ functional ability [26]. Moreover, it was reported that a complete and comprehensive assessment is the first step towards identifying a patient’s level of functional independence and rehabilitation needs and determining the effectiveness of treatment [27].

A few similar studies have explored the effect of additional CKC/balance exercises in addition to the typical post-TKA physiotherapy intervention and reported that the participants in the experimental groups demonstrated significantly greater improvement in the targeted outcomes [5,27-28]. In the study of Piva et al., improvement seemed greater for gait speed, single-leg stance time, and stiffness in the group where a supplementary CKC/balance exercise program was implemented, in both post-intervention (8 weeks) and follow-up (end of the 6th month) measures [5]. In the study by Liao et al., patients in the experimental group exhibited significantly greater improvements in functional reach test (p<0.01), single-leg stance time, gait speed, TUG, and sit-to-stand ability (p<0.05) at the eighth postoperative week and at the end of the fourth postoperative month [27]. In the study by Jogi et al., patients who participated in the experimental group demonstrated significantly greater improvement in the BBS and the TUG tests (p<0.01) at the 6th postoperative week [28]. The aforementioned studies had a relatively short follow-up period and no information is available on whether the results were maintained over a longer time. In our study, the CKC additional intervention started earlier after surgery (on the 5th postoperative day) compared with the other studies, and the implementation of the additional CKC exercises gave participants in the CKC-PT group greater functional improvement and balance confidence compared to the TKA-PT group, even 12 months after TKA [5,27-28].

Our additional CKC program did not affect the incidence of postoperative falls. This fact could probably be explained by the limited duration of the additional CKC program (5 weeks). A Cochrane Database review reported that a combined strength/balance and functional retraining exercise offered greater functional improvement, as well as reducing the frequency of falls, but the additional intervention programs in the included studies lasted from a minimum of eight weeks to a maximum of six months [29].

In reﬁning postoperative physiotherapy intervention, it is important to consider factors that may contribute to patients’ functional deﬁcits after TKA. One such factor may be impaired balance control. Although several tendons, the capsule, and remaining ligaments are retightened during TKA surgery, to restore the joint spaces damaged by the OA and to restore the intraarticular geometry, some of the knee’s ligaments are removed or released. These alterations may affect the function of several mechanoreceptors and cause impairment of postural control and balance. Several studies have identiﬁed deﬁcits in components of the balance system in patients who have undergone TKA, such as a decreased ability to detect joint position and motion, delayed muscle latency, altered amplitude of muscle activity, and decreased postural control [5]. The CKC exercises not only stimulate the co-contraction of the agonist and antagonist muscles to provide greater articular stability, but they also improve proprioception. The feedback is believed to be more efficient because of the impact of the body’s compressive forces and the contact of the foot with the ground. Furthermore, this type of exercise enhances the production of functional movements of the lower limb through concentric and eccentric contractions of the muscles involved in the joint movements of the hip, knee, and ankle that are commonly performed during daily activities [7]. Additionally, it was reported that the CKC exercises are able to improve the physical function of the elderly, allowing them to be more independent [8]. A positive factor that stands out is the contribution of these types of exercises to an improvement in bodily awareness that is reflected in body positioning control during activities and also helps with postural control [30]. Therefore, CKC exercises aimed at improving the impaired postural control and balance of patients after TKA should be considered.

Strengths and limitations

The present study was a prospective, interventional trial. The dropout rate was very low, for two reasons: first, following discharge, the rehabilitation program continued at the patient’s home, making it easy to comply; and second, patients who participated in the study received the physiotherapy treatment free of charge - an important factor in view of the current financial crisis in Greece. All participants underwent hybrid TKA through a medial parapatellar approach [18]. All procedures were performed by the same team of orthopedic surgeons and the implanted prostheses had similar specifications and technical characteristics. In addition, the same physiotherapist (TT) was responsible for the physiotherapy intervention in both groups. Supervision and guidance from the physiotherapist during sessions helped ensure the patient’s adherence. Furthermore, all measurements were made by the same examiner, who was not involved in any part of the rehabilitation program and was blinded with respect to the group assignment. These factors added strength and statistical power to the results of this study.

On the other hand, there are important limitations that must be mentioned. The most important was the limited duration of the intervention. Moreover, the study’s randomization was not computerized (block, stratified, or covariate adaptive randomization). However, the quasi-random procedure used is an acceptable randomization method that ensures that investigators cannot change who gets the next assignment, thus biasing the measurement of the effects of the interventions [17]. In addition, because of limited equipment availability, it was not possible to record knee muscle strength measurements such as isometric or isokinetic strength, maximum torque, endurance, or total work. Finally, the study’s sample population consisted of individuals who were capable of performing exercises in an upright position. Therefore, it must be underlined that our findings cannot be generalized to all knee OA patients.

## Conclusions

The results shown here indicate that the implementation of a CKC exercise program, in addition to the standard TKA-PT intervention, may significantly increase both the functional ability and the balance conﬁdence of patients with knee OA who have undergone TKA. Further research using randomized controlled trials is needed to validate the current outcomes in a larger number of patients and to thoroughly explore the effects of CKC exercises on functionality and balance after TKA.

## Appendices

**Table 5:** Physiotherapy for patients who have undergone total knee arthroplasty Recommendation Notes: During the first postoperative days, a towel roll should be placed under the ankle of the operated limb to promote knee extension when patients are supine in bed. During the first postoperative days, ensure that patients are premedicated with pain medication 30-60 minutes prior to each physiotherapy session and encourage them to use cryotherapy at least five times/day. Until the seventh postoperative week, cryotherapy is also recommended following physiotherapy sessions to reduce pain, discomfort, and swelling in the knee joint. All patients are educated to breathe with a normal inhalation/exhalation rhythm while performing the isometric exercises, in order to avoid a Valsalva maneuver. Isometric contractions should follow the BRIME (BRief Isometric Exercise) protocol suggested by Liberson et al, which consists of 6 s of isometric contraction, followed by 20 s of rest (purported to maintain normal pulse and blood pressure. Monitor wound healing and consult with the orthopedic surgeon if signs or symptoms of infection are seen. If the patient has any difficulty with a particular exercise, the program can be modified accordingly. Each exercise – apart from ankle pumps – starts with one set (5 to 10 repetitions), reaching a maximum of two sets of 10 repetitions, as individually tolerated. It is strongly recommended that exercises involving a risk of falling should be performed only under the physiotherapist’s supervision.

Physiotherapy for Patients Who Have Undergone Total Knee Arthroplasty (TKA-PT)			
Intervention	Performance Guidelines		
Timeframe: 1^st ^week (Inpatient Rehabilitation)			
Respiratory Physiotherapy (If needed)	Diaphragmatic breathing. Exhale and cough instructions must be given for secretion drainage. Upper limb combined with respiratory exercises can be performed.		
Trunk Exercises (If needed)	Supine position. Trunk extensors, individually modified abdominal exercises, pelvic elevation (pelvic bridge), etc.		
Isotonic and resistive training of the contralateral limb (If needed)			
Ankle Pumps	Supine position. Two sets of 10 repetitions each for both limbs, alternately. (Note: Be alert for any signs of deep vein thrombosis, such as increased swelling, erythema, calf pain)		
Ankle dorsiflexion – plantar flexion with manual resistance			
Isometric contractions of hip extensor muscles	Supine position. Isometric contraction for 6 s followed by a rest interval of 20 s.		
Active isotonic contractions of hip rotators	Supine position with the knee joint in extension and ankle joint in dorsal flexion.		
Hip abduction – adduction slides	Supine position. Neutral leg position (concerning hip rotation), with the knee joint in extension and ankle joint in dorsal flexion. During the first postoperative days, the exercise is performed with assistance, if needed, later as active isotonic.		
Lower limb stretching exercises	Mild stretching of hip flexors, extensors and adductors, knee extensors, and dorsiflexors.		
Activation of Knee’s Extensor Mechanism			
Isometric contractions of knee extensors	Supine position. Isometric contraction for 4 s, with adequate resting intervals. Gain knee extension equal to 0˚		
Isotonic knee extension (short arc)	Supine position. A towel roll is placed under the patient’s knee so that in the initial position the knee joint is 30˚ flexed. The patient is asked to straighten the lower leg, lift the foot and extend the knee. The patient must hold and count to 5-6, then lower the foot slowly with the ankle joint in the dorsal flexion position.		
Straight leg raise (SLR)	Supine position with the contralateral limb’s knee flexed and the foot supported on the bed. During the first postoperative days, patients are encouraged to perform the exercise even if the extensor mechanism of the knee joint has not fully recovered. Ultimately, as the program progresses with intensive activation of the extensor mechanism, the patients should be able to achieve SLR using full knee extension with strong quadriceps activation. The exercise can be started with the isometric holding of the limb at a hip angle of 50-60˚, followed by eccentric contraction of the hip flexors, and completed with the concentric elevation of the limb with the knee fully extended.		
Knee Flexion			
Stretching and range of motion exercises		Seated position on the lateral side of the bed, with the contralateral foot supported on a footstool. During the first postoperative days, the exercise is performed with assistance; later on, active isotonic contractions of hamstrings are performed, gradually increasing the range of knee flexion ( ≥70˚ on 5^th^ postoperative day)	
Isometric – isotonic strengthening of knee flexors		Supine position. Isometric contraction for 6 s followed by a rest interval of 20 s.	
Transfer Training	Patients are educated to perform bed mobility and transfers from bed to chair with the least amount of assistance while maintaining appropriate weight-bearing precautions as individually tolerated.		
Gait Training	The correct pattern of postoperative gait is taught according to three-point ambulation: walker-operated limb – contralateral limb. (Note: Avoid torsion forces across the knee joint, especially when bearing weight on the operated limb)		
Training of ascent/descent stairs (If patient ambulated with walker without any other assistance)	Before discharge, the patient is trained to ascend/descend stairs using the stair handrail and a forearm crutch. During ascent the contralateral limb is moved first, followed by the operated limb and by the crutch. During descending the crutch is placed on the front stair, followed by the operated limb and then by the contralateral limb. (Note: Avoid torsion forces across the knee joint, especially when bearing weight on the operated limb)		
Knee range of motion timeframe goal	The goal is to gain a pain-free range of knee motion from 0˚ (full extension) up to 90˚(knee flexion) 7 to 10 days after surgery		
Timeframe: 2^nd^ - 4^th^ weeks (Outpatient rehabilitation)			
The 1^st^ week’s activities and exercises are continued and developed	Individualized progressive improvement of muscle strength, endurance (isometric à isotonic à resistive training)		
Lower limb stretching exercises	Stretches of all muscle groups involved.		
Patella-femoral joint mobilization	Patella-femoral joint mobilization as indicated, taking account of incision healing.		
Activation of Knee’s Extensor Mechanism			
Isometric contractions of knee extensors	Supine position. Isometric contraction for 4 s, with adequate resting intervals.		
Isotonic knee extension (long arc)	Seated position on a chair with armrests and seat height 45-50 cm. The patient extends the knee joint (90˚→ 0˚). The patient must hold and count to 5, then lower foot slowly with the ankle in the dorsal flexion position.		
Knee Flexion:			
Seated heel slides	Seated position on a high chair so that the feet barely touch the floor. The patient flexes the knee joint to achieve an active range of motion >90˚.		
Balance exercises from a short-sitting position on the bed	Seated position on the side of the bed, with the hips in abduction and the feet resting on the floor. The patient performs trunk and upper-limb exercises.		
Bodyweight transfers from contralateral to operated lower limb	Upright position. The patient’s lower limbs are positioned in slight hip abduction with the back supported against the wall. The walking device is placed in front to provide a handhold for support. Initially, the load on the operated limb should be a maximum of 30% vs 70% on the contralateral. By the end of the 4^th^ week, the patient should be able to perform the exercise with an equal load on both limbs (50% vs 50%), without being supported by the wall and while maintaining the correct trunk and pelvic posture, always under the physiotherapist’s supervision.		
Standing toe raises	Upright position. When the patient acquires sufficient balance, can easily change direction during walking with assistance, and is able to turn safely while walking with the assistive device, training with exercises in the upright position may be commenced. The hands hold on to a firm surface to provide the patient with support.		
SLR in 4 planes (abduction, adduction, flexion, hyperextension)			
Gait training	Gait training is continued in order to improve the function and quality of operated limb performance during the swing and stance phases. The patient is re-educated so that during the initial contact of the operated limb’s stance phase the heel strikes the ground first, as in a normal gait pattern. After the 4^th^ week, if the patient’s postoperative muscle performance is adequate for weight-bearing acceptance, the walker is replaced by two forearm crutches or one cane, according to the surgeon’s preference.		
Knee range of motion timeframe goal	The goal is to gain a pain-free range of knee motion from 0˚ (full extension) to 105-110˚ (knee flexion)		
Timeframe: 5^th^ - 8^th^ week			
Lower limb stretching exercises	Emphasis on soft tissue stretching and joint flexibility.		
Sit-to-stand exercise	Chair with armrests and seat height 45-50 cm. The patient is seated with the knee joint at 90˚. The patient rises from the chair by pushing off both armrests and then slowly sits down again. (Note: The patient must have regained an active range of knee motion of at least 80˚ to perform sit-to-stand transfers with minimal compensatory activity)		
Forward Lunges 1/4	Upright position. A sturdy chair is placed on the contralateral side to provide the patient with handhold support. Keeping the back straight, the patient takes a small step forward with the operated limb, bending the operated knee.		
Mini squats (knee flexion/extension)	Upright position with patient’s back and shoulders against a wall. The feet are placed at shoulder's width apart, about 15-20 cm from the wall, with the toes pointing straight ahead. From the upright position (knee extension 0˚) the patient performs knee flexion up to 50-60˚ and then returns to upright with the legs straight. (Note: The patient is educated to perform the exercise with 40% weight on the operated limb and 60% on the contralateral limb).		
Standing toe raises	Upright position. The degree of difficulty depends on how the patient is supported while performing the exercises. Initially, the patient uses both hands, then just the hand on the operated side, and is ultimately supported using only two fingers on the operated side.		
SLR in 4 planes (flexion, abduction, adduction, extension)			
Lateral steps	Upright position. The patient is facing forward and performs lateral steps, starting from the operated side (initially 5 steps are performed for each side).		
Balance and proprioceptive training	Standing with eyes open and then closed. Standing with one foot directly in front of the other. Stepping forward/backward, etc., as individually tolerated.		
Knee range of motion timeframe goal	The goal is to preserve knee extension 0˚ and gain knee flexion >110˚.		
Timeframe: 9^th^ - 12^th^ week			
Lower limb stretching exercises			Emphasis on soft tissue stretching and joint flexibility. Maximize postoperative range of motion (-10˚ to 115˚ plus)
Sit-to-stand exercise			Chair with armrests and seat height 45-50 cm. The patient rises from the chair by pushing off both armrests and then slowly sits down again.
Knee extension resistive exercises			Sitting position. The exercises are performed with cuff-weights (Note: The cuff-weight resistance training program gradually increases in difficulty, with the addition of 0.5 kg increments up to a maximum of 2.5 kg, as individually tolerated)
Knee flexion resistive exercises			
Lateral steps			Upright position. The patient is facing forward and performs lateral steps, starting from the operated side (initially 5 steps are performed for each side).
Step on an obstacle / forward step down			Increase height and/or width of obstacle, as individually tolerated.
Lateral stepping over obstacles			Starting from 5 obstacles to reach the maximum threshold of 10 obstacles.
Balance and proprioceptive training			Walking on uneven or soft surfaces, with eyes opened/closed, walking backward. Sitting on Swiss Ball, standing on BOSU, etc., as individually tolerated.
Ascent/descent stairs step-over-step (12^th^ week)			Criteria for step-over-step stair climbing Pain-free active range of knee motion At least 4+/5 muscular performance based on Muscle Manual Test of all operated lower extremity muscle groups.
